# The Incidence and Genetic Diversity of Apple Mosaic Virus (ApMV) and Prune Dwarf Virus (PDV) in *Prunus* Species in Australia

**DOI:** 10.3390/v10030136

**Published:** 2018-03-19

**Authors:** Wycliff M. Kinoti, Fiona E. Constable, Narelle Nancarrow, Kim M. Plummer, Brendan Rodoni

**Affiliations:** 1 Agriculture Victoria, AgriBio, La Trobe University, Melbourne VIC 3083, Australia; Fiona.constable@ecodev.vic.gov.au (F.E.C.); Narelle.nancarrow@ecodev.vic.gov.au (N.N.); Brendan.Rodoni@ecodev.vic.gov.au (B.R.); 2 School of Applied Systems Biology, AgriBio, La Trobe University, Melbourne VIC 3083, Australia; 3 Animal, Plant and Soil Sciences Department, AgriBio, La Trobe University, Melbourne VIC 3083, Australia; K.Plummer@latrobe.edu.au

**Keywords:** *Ilarvirus*, *Prunus*, amplicon high-throughput sequencing, *Apple mosaic virus* (ApMV), *Prune dwarf virus* (PDV), genetic diversity

## Abstract

Apple mosaic virus (ApMV) and prune dwarf virus (PDV) are amongst the most common viruses infecting *Prunus* species worldwide but their incidence and genetic diversity in Australia is not known. In a survey of 127 *Prunus* tree samples collected from five states in Australia, ApMV and PDV occurred in 4 (3%) and 13 (10%) of the trees respectively. High-throughput sequencing (HTS) of amplicons from partial conserved regions of RNA1, RNA2, and RNA3, encoding the methyltransferase (MT), RNA-dependent RNA polymerase (RdRp), and the coat protein (CP) genes respectively, of ApMV and PDV was used to determine the genetic diversity of the Australian isolates of each virus. Phylogenetic comparison of Australian ApMV and PDV amplicon HTS variants and full length genomes of both viruses with isolates occurring in other countries identified genetic strains of each virus occurring in Australia. A single Australian *Prunus* infecting ApMV genetic strain was identified as all ApMV isolates sequence variants formed a single phylogenetic group in each of RNA1, RNA2, and RNA3. Two Australian PDV genetic strains were identified based on the combination of observed phylogenetic groups in each of RNA1, RNA2, and RNA3 and one *Prunus* tree had both strains. The accuracy of amplicon sequence variants phylogenetic analysis based on segments of each virus RNA were confirmed by phylogenetic analysis of full length genome sequences of Australian ApMV and PDV isolates and all published ApMV and PDV genomes from other countries.

## 1. Introduction

*Apple mosaic virus* (ApMV) and *Prune dwarf virus* (PDV) are members of the genus *Ilarvirus* (family *Bromoviridae*), which have a positive-sense, single-stranded tripartite RNA genome organization composed of RNA1, RNA2, and RNA3 [1]. RNA1 and RNA2 encode for non-structural proteins P1 and P2, which contain a methyltransferase domain (MT) and polymerase domain (RdRP) respectively, and are involved in viral replication. RNA3 is bi-cistronic encoding for the movement protein (MP) and coat protein (CP) that is expressed via sub-genomic RNA4 [2,3]. The ApMV RNA1, RNA2, and RNA3 consist of 3476, 2979, and 2056 nucleotides (nts) respectively and PDV RNA1, RNA2, and RNA3 consist of 3374, 2593, and 1683 nts respectively [4,5,6].

ApMV and PDV infect a wide variety of herbaceous and woody hosts, including *Prunus* species, and can cause diseases of economic importance [7]. Symptom expression associated with either virus is highly variable and depends upon virus strain, host species, and cultivar and can also be affected by environmental factors, especially higher temperatures which can mask symptoms [8]. ApMV infection in many *Prunus* trees is characterised by yellow discolorations, line or oak-leaf patterns, and vein banding of leaves, but it may also be latent [9]. ApMV infection causes significant crop yield reduction of up to 25% in some commercial *Prunus* species [10]. PDV infection can induce a variety of symptoms which may include chlorosis, mosaic, ringspot symptoms on leaves, and stunting [7]. It may also latently infect plants. Amongst the *Prunus* infecting ilarviruses, PDV has the most significant economic impact with infections attributed to yield losses of up to 100% in some commercial crops [7,11]. ApMV and PDV are graft transmissible and they persist in the vegetatively propagated material of infected trees. ApMV is not pollen transmitted in *Prunus* trees [12,13] whereas PDV is pollen transmitted, which contributes to its widespread distribution [14].

Assessment of the genetic diversity of ApMV and PDV has mainly been based on the analysis of the CP gene of isolates from different hosts and geographic regions. Early comparisons of the CP gene of ApMV isolates from Korea identified three phylogenetic groups that were separated based on plant host [15]. More recent studies of ApMV isolate sequences from various world geographic origins proposed the existence of two to five phylogenetic groupings of ApMV isolate sequences with no correlation to host or geographic origin [16,17,18].

Similarly, several phylogenetic groups have been reported for PDV isolates whereby one study of isolates from Turkey has reported the existence of four distinct phylo-groups [19] while a recent study using the CP sequences of all PDV isolates published and available in GenBank indicated the presence of only two phylo-groups [2]. Earlier studies suggested a separation of PDV isolate groupings based on host but later studies could not relate phylogenetic groups to host or geographic origin [19,20,21].

ApMV and PDV are known to occur in Australia, however, their incidence in *Prunus* trees and genetic diversity is not known [22,23,24]. In this study, samples from different *Prunus* species were collected from five states in Australia and tested for ApMV and PDV using PCR. Amplicons from conserved gene regions of RNA1, RNA2, and RNA3 of each the positive samples were deep sequenced and compared to corresponding GenBank sequences to determine the number ApMV and PDV genetic strains occurring in Australia compared to isolates from around the world. Full length genomes of Australian ApMV and PDV isolates that were previously generated by metagenomic HTS were compared phylogenetically to all available ApMV and PDV full length genome sequences from GenBank and these phylogenetic inferences were compared to those from the conserved gene regions of RNA1, RNA2, and RNA3.

## 2. Materials and Methods

### 2.1. Plant Material and Total RNA Extraction

Leaf tissue samples from 127 *Prunus* trees were collected in spring (2014–2015) from five states in Australia (Table 1). Symptoms characteristic of virus infections were not noted at the time of sample collection. Total RNA was extracted from 0.3 g leaf tissue (fresh weight) of each sample using the RNeasy^®^ Plant Mini Kit (Qiagen, Hilden, Germany) as described previously [21].

### 2.2. Reverse Transcription Polymerase Chain Reaction (RT-PCR) Amplification

RT-PCR tests were carried out using the SuperScript III One-Step RT-PCR System with Platinum^®^ Taq (Invitrogen, Carlsbad, CA, USA) according to the manufacturer’s instructions except that the total reaction volume was 25 μL. The PCR products were analysed by electrophoresis in 1.5% agarose gels that were stained with SYBR^®^ Safe DNA gel stain (Invitrogen) for visualization.

For preliminary screening by RT-PCR the total RNA extract of each sample was tested for ApMV and PDV using previously published primers that were specific for RNA3 of each virus [25,26].

Total RNA extracts from the *Prunus* tree samples that tested positive for the RNA3 for ApMV and/or PDV were used for RT-PCR amplification of partial segments of the (MT) gene on RNA1, RdRp gene on RNA2, and CP gene on RNA3 of both viruses using primers developed for this study (Table 2). Cycling conditions consisted of: a reverse transcription step at 48 °C for 45 min; denaturation at 94 °C for 2 min, followed by 35 cycles, denaturing at 94 °C for 30 s, annealing for 30 s at the appropriate temperature for each primer pair (Table 2), elongation at 72 °C for 1 min; and a final elongation step at 72 °C for 10 min.

### 2.3. Amplicon High-Throughput Sequencing (HTS) Library Preparation and Sequence Reads Analysis

The ApMV and PDV RNA1, RNA2, and RNA3 RT-PCR amplicons were gel purified using the Promega Wizard^®^ PCR clean-up kit (Promega, Madison, WI, USA) according to the manufacturer’s instructions. Amplicon libraries were prepared and sequenced using the Illumina MiSeq as described previously [27]. The sequence read data for this study have been submitted to the NCBI Sequence Read Archive (SRA) database under the Bioproject accession PRJNA404016 and SRA study accession SRP117219.

The generated raw amplicon sequence reads were quality trimmed, paired, and amplicon sequence reads that had a reverse orientation were reversed and complemented as previously described [27]. The amplicon reads were aligned using Muscle (version 3.8.31) [28] with default parameters. The overlapping alignment coverage for each RNA amplicon read was identified and Cutadapt (version 1.4.1) [29] with default parameters was used to trim the amplicon reads to the following lengths: 173, 364, and 452 nt for ApMV RNA1, RNA2, and RNA3 respectively; and 193, 358, and 396 nt for PDV RNA1, RNA2, and RNA3 respectively. Shorter reads in each set of amplicons were discarded. The trimmed amplicon sequence reads were then clustered at 100% identity and cluster sequences with less than 10 reads and non-coding cluster sequences filtered out of each RNA amplicon sample, as previously described [27].

### 2.4. Phylogenetic and Sequence Identity Analysis

Nucleotide sequences of RNA1, RNA2, and RNA3 for ApMV and PDV isolates from various *Prunus* species and geographical regions were retrieved from GenBank (Table S1) and trimmed to the corresponding region of the genome that was amplified from each of the ApMV and PDV isolates in this study. Sequence clusters of each amplicon generated were pooled separately with nucleotide sequences from each viral RNA component and aligned using Muscle (version 3.8.31) [28]. Maximum likelihood phylogenetic trees were constructed in RAxML (version 8.0.19) [30] using the GTRGAMMA model with 1000 bootstrap replicates and the resulting trees were visualized in FigTree (version 1.4.2) [31]. Branches that had less than 50% bootstrap support were collapsed. Sequence identity analysis using the sequence demarcation tool (SDT) (version 1.2) [32] was carried out on the aligned amplicon clusters of each viral RNA segment.

Full length genome sequences of the Australian ApMV isolate K75 and PDV isolates NS9 and PCH4 generated by metagenomic HTS in a previous study [33], were aligned with the full genome sequences of other ApMV and PDV isolates available in GenBank using Muscle (Table S2) (version 3.8.31) [28]. Maximum likelihood phylogenetic trees were constructed in MEGA (version 6) [34] with 1000 bootstrap replicates and the resulting trees were visualized in FigTree (version 1.4.2) [31]. Branches having less than 50% bootstrap support were collapsed.

## 3. Results

### 3.1. RT-PCR Detection of ApMV and PDV

ApMV and PDV were detected in five and 13 of the 127 *Prunus* tree samples respectively, using the published RT-PCR tests for each virus. ApMV was detected in three almond and two plum trees in New South Wales and Victoria. PDV was detected in three almonds, one apricot, eight peach, and one plum tree from four states in Australia (Table 3).

### 3.2. Amplicon Next Generation Sequencing Data and Read Cluster Analysis

The total number of raw reads obtained from amplicon HTS for RNA1, RNA2, and RNA3 of ApMV were 186,987, 111,256, and 196,503 reads respectively (Table S3). After quality trimming, the overall total number of reads used for analysis for ApMV RNA1, RNA2, and RNA3 was reduced to 172,748, 101,747, and 183,473 respectively (Table S3). For PDV the total number of raw reads obtained from amplicon HTS were 327,564, 345,823, and 628,407 for RNA1, RNA2, and RNA3 reads respectively and these were reduced to 300,465, 315,263, and 584,209 for RNA1, RNA2, and RNA3 respectively after quality trimming (Table S4).

The quality trimmed amplicon reads were clustered at 100% identity resulting in an average of 1090, 1495, and 2517 unique ApMV sequence variants per sample for RNA1, RNA2, and RNA3 respectively (Table 4). There was an average of 1061, 1466, and 2309 unique PDV sequence variants for RNA1, RNA2, and RNA3 respectively (Table 5). Filtering of the non-coding and sequence variants with less than 10 reads decreased the average number of ApMV sequence variants per plant sample to 122, 242, and 409 for RNA1, RNA2, and RNA3 amplicons respectively (Table 4), and to 124, 185, and 272 PDV sequence variants per plant sample for RNA1, RNA2, and RNA3 amplicons respectively (Table 5).

### 3.3. Phylogenetic and Sequence Identity Analysis

**Figure 1:**
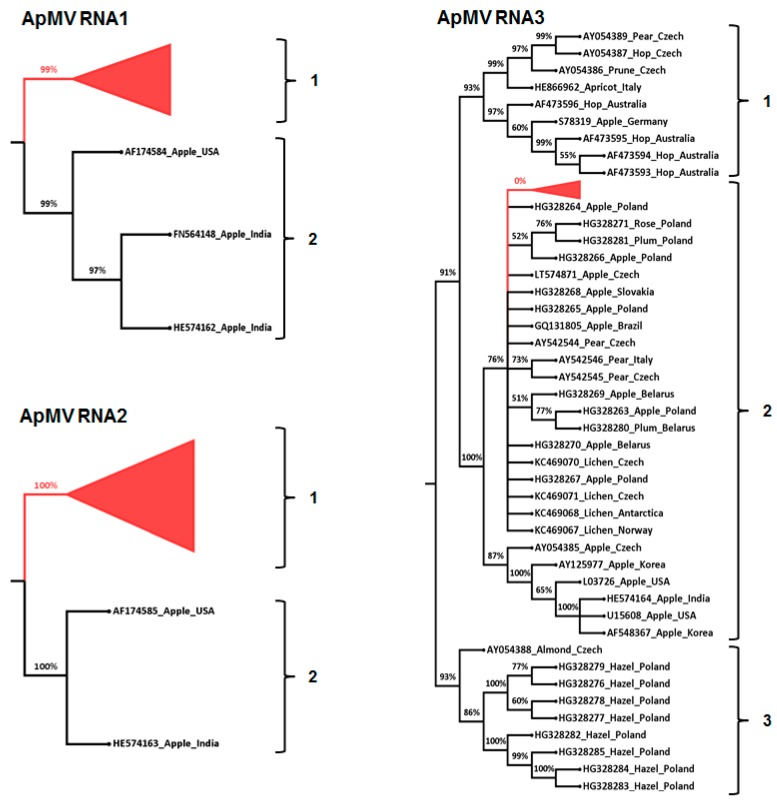
Maximum likelihood phylogenetic relationship of 608, 1210, and 2047 *apple mosaic virus* ApMV sequence variants of partial methyltransferase (MT), RNA dependent RNA polymerase (RdRp) and coat protein (CP) genes segments on RNA1, RNA2 and RNA3 respectively, from Australian *Prunus* trees and corresponding ApMV sequences from GenBank (Table S1). Branches having less than 50% bootstrap support were collapsed and the Australian sequence variants from this study were also collapsed for ease of presentation (red colour). Each of ApMV RNA1, RNA2, and RNA3 phylo-groups were supported by branches with >90% bootstrap, as indicated on the phylogenetic tree.

Phylogenetic analysis of each of the pooled ApMV sequence variants for each of RNA1, RNA2, and RNA3 and their corresponding published GenBank isolate sequences (Table 4; Table S5) provided >90% bootstrap support for two phylogenetic groups (phylo-groups 1 and 2) for each of RNA1 and RNA2 and three phylogenetic groups for RNA3 (phylo-groups 1, 2, and 3) (Figure 1). ApMV phylo-group 1 of RNA1 and phylo-group 1 of RNA2 each consisted of amplicon sequence variants from Australian *Prunus* trees only. Phylo-group 2 of RNA1 and phylo-group 2 of RNA2 each consisted of ApMV sequences occurring in apple plant hosts from other countries (Figure 1; Table S5). All Australian ApMV RNA3 amplicon sequence variants from this study occurred in phylo-group 2, which also consisted of sequences from 12 apples, 3 pears, 1 rose, and 4 lichen isolates occurring in other countries (Figure 1; Table S5). ApMV RNA3 phylo-group 1 consisted of sequences from four Australian hop isolates and also one isolate each from hop, prune, apricot, pear, and apple occurring in other countries. ApMV RNA3 phylo-group 3 contained sequences of four hazelnut isolates; two apple isolates and one isolate each from almond and strawberry (Figure 1; Table S5). None of the Australian ApMV isolates from this study occurred in RNA3 phylo-groups 1 or 3.

**Figure 2:**
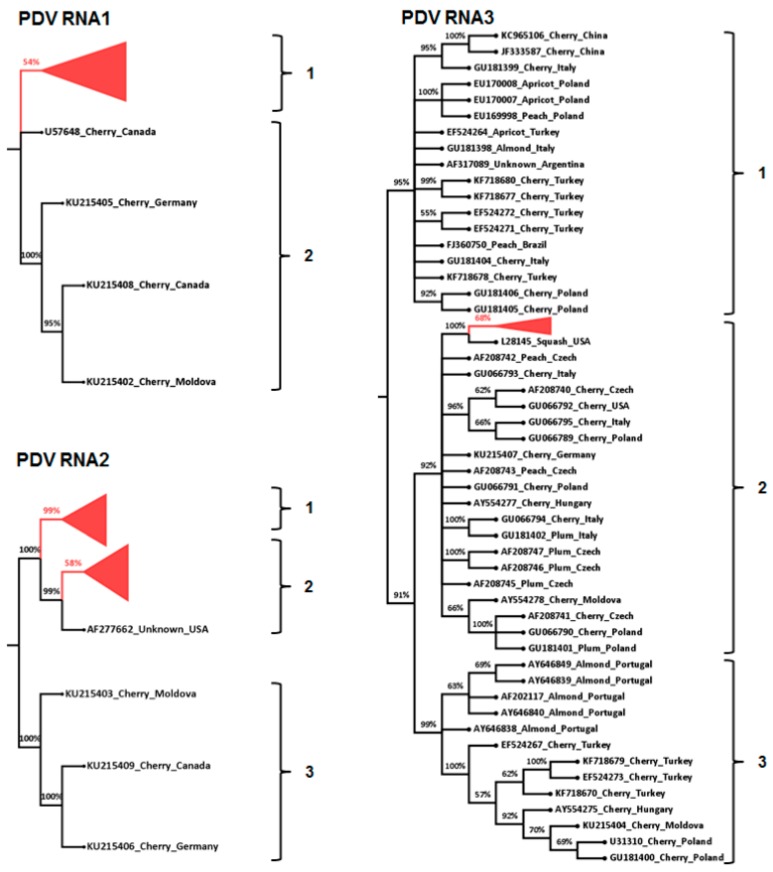
Maximum likelihood phylogenetic relationship of 1615, 2411, and 3232 *prune dwarf virus* (PDV) sequence variants of partial methyltransferase (MT), RNA dependent RNA polymerase (RdRp) and coat protein (CP) genes segments on RNA1, RNA2 and RNA3 respectively, from Australian *Prunus* trees and corresponding PDV sequences from GenBank (Table S1). Branches having less than 50% bootstrap support were collapsed and the Australian sequence variants from this study were also collapsed for ease of presentation (red colour). Each of PDV RNA1, RNA2, and RNA3 phylo-groups were supported by branches with >90% bootstrap, as indicated on the phylogenetic tree.

Phylogenetic analysis of PDV RNA1 sequences resulted in two phylo-groups with >90% bootstrap support and all Australian amplicon sequence variants from this study occurred in phylo-group 1 as well as a single cherry isolate from Canada. PDV RNA1 phylo-group 2 consisted of only three cherry isolates. PDV RNA2 had three phylo-groups and phylo-group 1 consisted only of Australian PDV sequence variants from this study, phylo-group 2 consisted primarily of Australian isolates but also included a single isolate from the USA. RNA2 sequence variants of Australian isolate CNS3 occurred in both phylo-group 1 and 2 (Figure 2; Table S5). Phylo-group 3 contained cherry isolates from various geographic origins, but none were from Australia. PDV RNA3 had three phylo-groups with sequence variants from this study only occurring in phylo-group 2 and which were most closely related to an experimental isolate maintained in squash from the USA. PDV RNA3 phylo-group 1 contained sequences from almond, apricot, cherry, and peach whereas phylo-group 3 contained isolates from only almond and cherry (Figure 2; Table S5).

SDT identity analysis indicated that amplicon variants of ApMV and PDV occurring within the same phylo-group for each of RNA1, RNA2, and RNA3 shared more than 97% sequence similarity except for PDV isolate CNS3. Amplicon sequence variants from this isolate had RNA2 variants occurring in two phylo-groups, 1 and 2. Each of the RNA 1 and 3 variants in this isolate and the RNA1, RNA2, and RNA3 variants of all other ApMV and PDV isolates only occurred in one phylogenetic group. (Table S6).

**Figure 3:**
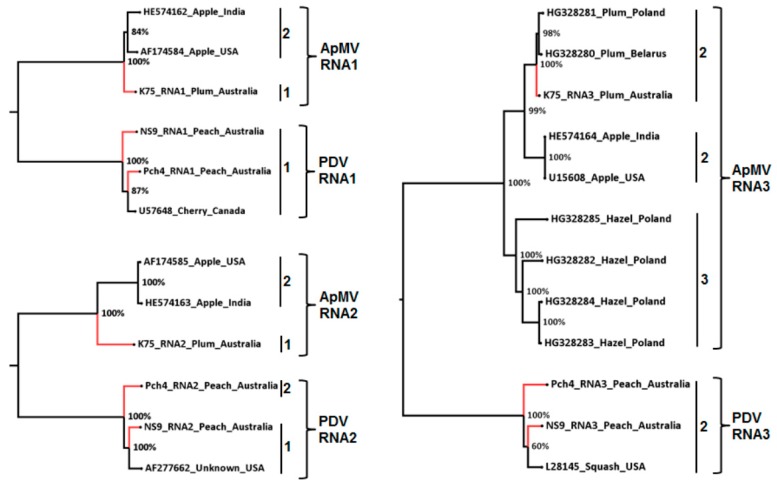
Maximum likelihood phylogenetic relationship of full-length RNA1, RNA2, and RNA3 genome sequences of Australian *apple mosaic virus* (ApMV) and *prune dwarf virus* (PDV) isolates and the corresponding full-length sequences of ApMV and PDV from the GenBank (Table S2). The branch positions of the Australian ApMV and PDV isolates are indicated in red colour. Each phylo-group corresponding to phylo-groups determined from phylogenetic analysis of ApMV and PDV amplicon sequence variants (Figure 1 and Figure 2) are numbered.

Phylogenetic analysis of the full-length ApMV RNA1 and RNA2 genome segments resulted in similar phylo-groupings to the phylogenetic analysis of their corresponding amplicon sequences. The Australian ApMV isolate K75 did not cluster with isolates from the USA and India, which clustered together. Three phylo-groups were observed from phylogenetic analysis of the full-length ApMV RNA3 which separated based on host and the Australian ApMV plum isolate occurred in plum group (Figure 3). The RNA1, RNA2, and RNA3 full-length genome sequences of both Australian PDV isolates occurred in the same phylogenetic groupings that were observed in the phylogenetic analysis of amplicon sequence variants (Figure 3).

## 4. Discussion

This study presents the first in-depth analysis of the incidence and genetic diversity of ApMV and PDV strains occurring in Australian *Prunus* trees. The low incidence of ApMV (3%) observed amongst the 127 Australian *Prunus* tree samples is similar to findings of several studies in other countries [2,35,36,37]. PDV only occurred in 10% of the Australian *Prunus* tree samples, which is in contrast to PDV survey studies from other countries that found a higher incidence, up to 40%, of PDV [2,35,36,37]. In Australia, many plantings of *Prunus* trees were established with virus-tested material, which would minimize virus transmission through vegetative propagation and pollen [12,38], and this could explain the low incidence of both ApMV and PDV.

Previous studies of ApMV and PDV diversity in other countries have focussed only on the MP and/or CP gene on RNA 3 [16,17,21,39]. This study provides a more comprehensive analysis of diversity of these viruses by using conserved gene regions of RNA1 and RNA2 in addition to RNA3 and also a comparison with full length genome sequences of these two viruses. Australian ApMV and PDV amplicon sequence variants and full length genome sequences of RNA1, RNA2, and RNA3 from *Prunus* trees clustered into distinct phylo-groups that were separate from previously published sequences from other hosts and/or geographic regions. The phylogenetic analysis identified a lower level of diversity within and between Australian isolates of each virus compared to the diversity observed amongst Australian PNRSV isolates that were also detected in *Prunus* trees [27]. Although only a limited number of full length sequences were available for RNA1, RNA2, and RNA3 of each virus, the phylogenetic analysis of these sequences also supported the phylo-groupings based on the amplicon sequences that were observed in this study.

A comparison of the amplicon sequences of Australian isolates with overseas isolates indicated that ApMV RNA1 and RNA2 each had two phylo-groups and ApMV RNA3 had three phylo-groups. Amongst early studies there was no clear consensus on the number of ApMV RNA3 phylogenetic groups and three or five phylo-groups were identified [15,16]. A recent phylogenetic study by Grimová, Winkowska, Ryšánek, Svoboda, and Petrzik [17] of ApMV isolates identified two major phylo-groups that were also observed by later studies [18]. In this study, these two phylo-groups were also observed, corresponding to phylo-groups 1 and 2, and a third phylo-group was also observed, consisting of recently published ApMV isolates from hazelnut that were not used in the earlier studies [40]. This study used representative sequences of phylogenetic clusters and sub-clusters from all these studies that had phylogenetically informative sites ensuring our study is the most current and inclusive analysis of ApMV RNA3 genetic diversity.

Similar to previous phylogeny studies on ApMV CP gene [17], there was no clear correlation between RNA3 phylo-groups to geographical origin in this study. However, the ApMV RNA3 phylo-groups identified in this study appeared to differentiate with host: phylo-groups 1 and 3 consisted mainly of, but were not limited to, isolates of hops and hazelnut respectively, whilst phylo-group 2 was associated with apple, lichen, pear, and plum. Similar RNA3 CP gene host based phylo-groupings have been previously reported and suggest that ApMV strains may have co-evolved with their plant host species resulting in CP gene diversity from host-imposed selective pressure [15]. This may be further supported by the observed host-based RNA3 phylogenetic groupings of full length genome segments of ApMV in this study, although only nine sequences were analysed.

Australian ApMV amplicon HTS sequence variants and full length genome sequences of RNA1 and RNA2 from Australian *Prunus* trees in this study were clustered into distinct phylo-groups and were separate from previously published sequences, which were mainly from apple trees and other geographic regions. It is difficult to ascertain if the separation of Australian isolates, which were all from *Prunus* species, from apple isolates occurring in other regions was based on host or geography due to limited availability sequences of ApMV isolates for comparative analysis.

The results of this study indicate that PDV had two RNA1 phylo-groups and three phylo-groups each for RNA2 and RNA3. RNA1 amplicon HTS sequence variants of the Australian PDV isolates occurred only in phylo-group 1 together with a Canadian PDV cherry isolate and this result was also supported by phylogenetic analysis of PDV RNA1 full length sequences. RNA1 phylo-group 2 comprised of three cherry isolates from diverse geographical origins. These results suggest that there may not be any correlation in PDV RNA1 phylo-groupings to host species or geographical origin. PDV RNA2 had three phylo-groups supported by phylogenetic analysis of partial and full length RNA2 sequences. Australian PDV isolates occurred in RNA2 phylo-groups 1 and 2, and a single Australian isolate (CNS3) had its sequence variants occurring in PDV RNA2 phylo-groups 1 and 2. This occurrence of virus sequence variants from a single isolate in multiple phylo-groups has been previously reported for the *Ilarvirus* PNRSV [27]. The biological implication of this occurrence of multiple distinct populations of PDV RNA2 in isolate CNS3 is not known, and it is possible that two separate infection events—either through grafting or by pollen—resulted in this dual infection.

The occurrence of the three PDV RNA3 phylo-groups in this study concurs with previous diversity analysis of PDV isolates from various host and geographical origin [2,19,21]. Previous studies reported that the PDV RNA3 CP gene groupings were based on host species or geographical origin [19,41], while other studies and this study could find no association [2,21,42]. In this study all Australian PDV isolates and all their sequence variants from different *Prunus* species occurred in phylo-group 3. The host or geographical origin specific groupings reported in other countries may have occurred due to the analysis of a high number of a single strain of PDV isolates sampled from multiple plants of a single host species in a particular geographic region. Similarly, no host or geographical origin specific groupings were observed from PDV RNA3 full length sequences phylogenetic analysis, although only three sequences were analysed. It is possible that greater PDV diversity exists in Australia, which might have been identified had more isolates been analysed.

The RNA1 and RNA2 phylo-groups of ApMV and PDV are based on limited sequences from other regions and very few hosts in Australia. More data is required to determine if these phylo-groups are correct. Similarly, the more data that becomes available for RNA3 of both viruses from different regions and hosts will further support the delineation of phylo-groups observed in this study. This information might then be used to study specific nucleic acid and protein motifs that are important in the interaction between hosts and viral strains.

The low genetic diversity of Australian ApMV and PDV strains observed in this study could indicate that they were derived from a single virus population or very few introductions on infected planting material into Australia, which may be associated with Australia’s strict quarantine regulations. However, only a few sequences from *Prunus* trees were available for comparison and analysis of a larger number of isolates from within Australia and in other countries from *Prunus* and other hosts is required to better understand the effect of host and geographic origin on strain differentiation and diversity.

Sequence variants occurring in the same phylo-group in each of the Australian ApMV and PDV RNA1, RNA2, and RNA3 had identities ranging from 97–99%. These findings are similar to the identity demarcation observed on a study of the *Ilarvirus* PNRSV by [27] who proposed that a genetic strain of PNRSV in a biological isolate (plant) must comprise of at least one variant of each RNA component that encodes the expected open reading frame (ORF); and may include sequence variants that are ≥97% similar. Extrapolating this definition to ApMV and PDV, this study identified a single *Prunus* infecting genetic strain of ApMV based on the occurrence of all ApMV isolates sequence variants occurring in a single phylo-group in each RNA: RNA1 phylo-group 1, RNA2 phylo-group 1 and RNA3 phylo-group 3. Based on the combination of RNA1, RNA2, and RNA3 phylo-groups, two genetic strains of PDV were identified to occur in Australia with one *Prunus* tree (CNS3) having both strains of PDV. The PDV strains were: RNA1 phylo-group 1, RNA2 phylo-group 1, and RNA3 phylo-group 2; and RNA1 phylo-group 1, RNA2 phylo-group 2, and RNA3 phylo-group 2.

The observed phylo-groups and proposed genetic strains of ApMV and PDV based on the amplicon HTS analysis was supported by the full genome phylogeny. This highlights the potential of amplicon HTS in achieving accurate phylogenetic inference based on analysis of a segment of a virus genome and also to detect mixed populations of virus strains. However, the use of complete genomes to define phylogenetic groupings may give a clearer insight on correlation of such groupings to host species or geographic origin due to the larger volume of sequence information.

## Figures and Tables

**Table 1:** The location and the number of each *Prunus* species used in this study.

Location	*Prunus* Species	No. of Samples
New South Wales	Almond (*P. dulcis*)	39
Queensland	Apricot (*P. armeniaca*)	4
	Nectarine (*P. persica var. nucipersica*)	1
	Plum (*P. domestica*)	6
	Peach (*P. persica*)	6
	Sweet Cherry (*P. avium*)	2
South Australia	Nectarine (*P. persica var. nucipersica*)	3
	Peach (*P. persica*)	1
Tasmania	Apricot (*P. armeniaca*)	1
	Almond (*P. dulcis*)	1
	Peach (*P. persica*)	5
	Plum (*P. domestica*)	2
	Sweet Cherry (*P. avium*)	8
Victoria	Almond (*P. dulcis*)	30
	Plum (*P. domestica*)	4
	Purple leaf plum (*P. cerasifera*)	2
	Peach (*P. persica*)	12

**Table 2:** Primers used for the RT-PCR amplification of RNA1, RNA2, and RNA3 segments of the apple mosaic virus (ApMV) and prune dwarf virus (PDV) genomes.

Primers	Primer Sequence (5’-3’)	RNA (Gene Target) ^a^	Amplicon Length	Annealing Temp.	Reference
APMV-MT1	AGTTTGTGTGATGTGAGAT	RNA1 (MT)	222 bp	53 °C	This study
APMV-MT2	ATTTCTAAGGCGTAACTTC				
APMV-Rd1	TCATTGGATCCCTTTGCTTC	RNA2 (RdRp)	383 bp	59 °C	This study
APMV-Rd2	AAACTCGTCGTCCCTATCC				
APMV-CP1	TTGCGTTAATTGCAAGTGG	RNA3 (CP)	471 bp	52 °C	This study
APMV-CP2	TCAAAAGTTGTGTTTGGAG				
PDV-MT1	GCGCTGACGAGACTACTA	RNA1 (MT)	205 bp	55 °C	This study
PDV-MT2	GCGAAACTGTGTGAGGAA				
PDV-Rd1	CGTTTCTGGAAGGAAGTGG	RNA2 (RdRp)	382 bp	60 °C	This study
PDV-Rd2	TTGCTTCGAAATTGAACAA				
PDV-CP1	TGTTAAGAAACAATTCCCA	RNA3 (CP)	422 bp	57 °C	This study
PDV-CP1	GCTGAAAAGCGTTGTCATA				

^a^ Gene targets encoding: MT = methyltransferase; RdRp = RNA-dependent RNA polymerase; CP = Coat Protein.

**Table 3:** *Prunus* tree samples that tested positive for apple mosaic virus (ApMV) and prune dwarf virus (PDV) and their location (state) of origin in Australia.

Isolate	Host	Location	Virus Detected
K73	Almond (*P. dulcis*)	Victoria	ApMV
K74	Plum (*P. domestica*)	Victoria	ApMV
K75	Plum (*P. domestica*)	Victoria	ApMV
M35	Almond (*P. dulcis*)	New South Wales	ApMV
M36	Almond (*P. dulcis*)	New South Wales	ApMV
CNS3	Peach (*P. persica*)	Victoria	PDV
CNS6	Peach (*P. persica*)	Victoria	PDV
K76	Almond (*P. dulcis*)	Victoria	PDV
NM20	Almond (*P. dulcis*)	New South Wales	PDV
NM21	Almond (*P. dulcis*)	New South Wales	PDV
NS3	Peach (*P. persica*)	Victoria	PDV
NS5	Peach (*P. persica*)	Victoria	PDV
NS7	Peach (*P. persica*)	Victoria	PDV
NS9	Peach (*P. persica*)	Victoria	PDV
PCH4	Peach (*P. persica*)	Victoria	PDV
Q1	Apricot (*P. armeniaca*)	Queensland	PDV
Q10	Plum (*P. domestica*)	Queensland	PDV
Tas6	Peach (*P. persica*)	Tasmania	PDV

**Table 4:** The number of reads generated from the next generation sequencing of the amplicons derived from the partial methyl transferase (MT) gene on RNA1, partial RNA dependent RNA polymerase (RdRp) gene on RNA2, and partial coat protein (CP) gene on RNA3 of apple mosaic virus (ApMV) and the number of sequence variants of each partial gene region before and after cluster analysis.

APMV	RNA1 (Partial MT Gene: 173 bp)			RNA2 (Partial RdRp Gene: 364 bp)			RNA3 (Partial CP Gene: 452 bp)		
Plant ID	No. of Reads after Trimming	No. of Sequence Variants	No. of Sequence Variants After Filter ^a^	No. of Reads after Trimming	No. of Sequence Variants	No. of Sequence Variants after Filter	No. of Reads after Trimming	No. of Sequence Variants	No. of Sequence Variants after Filter
K73	48,587	1042	123	20,384	1114	186	34,454	1784	298
K74	14,740	843	107	24,659	1532	242	62,852	2816	445
K75	51,711	818	126	19,328	1422	229	55,305	2335	376
M35	42,301	1516	141	16,127	1681	227	18,273	2585	349
M36	15,409	1231	111	21,249	1725	326	12,589	3063	579
Average	34,550	1090	122	20,349	1495	242	36,695	2517	409

^a^ Filtering of the non-coding and sequence variants with less than 10 reads.

**Table 5:** The number of reads generated from the next generation sequencing of the amplicons derived from the partial methyl transferase (MT) gene on RNA1, partial RNA dependent RNA polymerase (RdRp) gene on RNA2, and partial coat protein (CP) gene on RNA3 of prune dwarf virus (PDV) and the number of sequence variants of each partial gene region before and after cluster analysis.

PDV	RNA1 (Partial MT Gene: 193 bp)			RNA2 (Partial RdRp Gene: 358 bp)			RNA3 (Partial CP Gene: 396 bp)		
Plant ID	No. of Reads after Trimming	No. of Sequence Variants	No. of Sequence Variants after Filter ^a^	No. of Reads after Trimming	No. of Sequence Variants	No. of Sequence Variants after Filter	No. of Reads after Trimming	No. of Sequence Variants	No. of Sequence Variants after Filter
CNS3	16,020	1164	128	48,112	2101	270	56,440	2610	308
CNS6	18,060	1087	137	21,083	2870	386	19,377	1055	134
K76	20,460	1426	164	12,842	1409	185	16,638	1890	291
NM20	12,330	725	103	12,127	1234	142	25,249	1197	195
NM21	15,387	1043	121	16,178	1024	136	11,833	1640	182
NS3	26,188	1027	116	17,450	1436	163	17,205	2960	373
NS5	42,242	1191	137	25,272	985	135	85,141	3866	397
NS7	10,542	645	98	13,794	1237	152	81,229	2928	342
NS9	27,752	1080	121	31,725	1099	112	47,676	1916	251
PCH4	47,346	1075	115	22,097	1140	147	79,293	2805	359
Q1	30,758	1089	110	12,866	1038	136	26,150	1519	231
Q10	11,998	884	107	50,068	1159	131	23,349	1650	193
Tas6	21,382	1362	158	31,649	2324	316	94,629	3985	366
Average	23,113	1061	124	24,251	1466	185	44,939	2309	272

^a^ Filtering of the non-coding and sequence variants with less than 10 reads.

## Supplementary Materials

The following are available online at http://www.mdpi.com/1999-4915/10/3/136/s1, Table S1: The origin, host and the GenBank accession number of the apple mosaic virus (ApMV) and prune dwarf virus (PDV) RNA1, RNA2, and RNA3 sequences that were used for phylogenetic analysis in this study. Table S2: The origin, host, length of full genome sequence and the corresponding GenBank accession number of the apple mosaic virus (ApMV) and prune dwarf virus (PDV) RNA1, RNA2, and RNA3 genome segments that were used for phylogenetic analysis in this study. Table S3: The number of raw sequences reads generated from the next generation amplicon sequencing of apple mosaic virus (ApMV) methyltransferase (MT), RNA dependent RNA polymerase (RdRp) and coat protein (CP) partial gene segments on RNA1, RNA2, and RNA3 respectively and the number of reads remaining after trimming. Table S4: The number of raw sequences reads generated from the next generation amplicon sequencing of prune dwarf virus (PDV) methyltransferase (MT), RNA dependent RNA polymerase (RdRp) and coat protein (CP) partial gene segments on RNA1, RNA2, and RNA3 respectively and the number of reads remaining after trimming. Table S5: The phylo-groups identified from phylogenetic analysis of amplicon sequence variants from partial gene segments of methyltransferase (MT), RNA dependent RNA polymerase (RdRp) and coat protein (CP) genes on RNA1, RNA2 and RNA3 respectively of the Australian apple mosaic virus (ApMV) and prune dwarf virus (PDV) isolates and GenBank isolate sequences. Table S6: The % identity cut-off of apple mosaic virus (ApMV) and prune dwarf virus (PDV) amplicon sequence variants from partial gene segments of methyltransferase (MT), RNA dependent RNA polymerase (RdRp) and coat protein (CP) genes on RNA1, RNA2, and RNA3 respectively determined by sequence identity analysis.

## References

[1] Bujarski J., Figlerowicz M., Gallittelli D., Roossinck M., Scott S., King A.M.Q., Adams M.J., Carstens E.B., Lefkowitz E.J. (2012). Family bromoviridae. Virus Taxonomy, Ninth Report of the International Committee on Taxonomy of Viruses.

[2] Pallas V., Aparicio F., Herranz M., Amari K., Sanchez-Pina M., Myrta A., Sanchez-Navarro J. (2012). Ilarviruses of *prunus* spp.: A continued concern for fruit trees. Phytopathology.

[3] Codoner F.M., Elena S.F. (2008). The promiscuous evolutionary history of the family *bromoviridae*. J. Gen. Virol..

[4] Shiel P., Berger P. (2000). The complete nucleotide sequence of *apple mosaic virus* (ApMV) RNA 1 and RNA 2: ApMV is more closely related to *alfalfa mosaic virus* than to other *ilarviruses*. J. Gen. Virol..

[5] Rampitsch C., Eastwell K. (1997). The complete nucleotide sequenceof prune dwarf *ilarvirus* RNA-1. Arch. Virol..

[6] Shiel P., Alrefai R., Domier L., Korban S., Berger P. (1995). The complete nucleotide sequence of *apple mosaic virus* RNA-3. Arch. Virol..

[7] Nemeth M., Szalay-Marzsó L., Posnette A. (1986). Virus, Mycoplasma and Rickettsia Diseases of Fruit Trees.

[8] Wood G. (1979). Virus and Virus-Like Diseases of Pome Fruits and Stone Fruits in New Zealand.

[9] Šutić D.D., Ford R.E., Tošić M.T. (1999). Handbook of Plant Virus Diseases.

[10] Martelli G., Savino V. (1997). Infectious diseases of almond with special reference to the mediterranean area 1. Bull. OEPP.

[11] Çağlayan K., Ulubas-Serce C., Gazel M., Varveri C. (2011). Prune dwarf virus. Virus and Virus-Like Diseases of Pome and Stone Fruits.

[12] Fulton R. (1972). Apple Mosaic Virus. CMI/AAB Descriptions of Plant Viruses.

[13] Digiaro M., Savino V., Terlizzi B.D. (1992). Ilarviruses in apricot and plum pollen. Acta Hortic..

[14] Mink G. (1993). Pollen and seed-transmitted viruses and viroids. Annu. Rev. Phytopathol..

[15] Lee G.P., Ryu K.H., Kim H.R., Kim C.S., Lee D.W., Kim J.S., Park M.H., Noh Y.M., Choi S.H., Han D.H. (2002). Cloning and phylogenetic characterization of coat protein genes of two isolates of *apple mosaic virus* from ‘fuji’apple. Plant Pathol. J..

[16] Lakshmi V., Hallan V., Ram R., Ahmed N., Zaidi A., Varma A. (2011). Diversity of *apple mosaic virus* isolates in india based on coat protein and movement protein genes. Indian J. Virol..

[17] Grimová L., Winkowska L., Ryšánek P., Svoboda P., Petrzik K. (2013). Reflects the coat protein variability of *apple mosaic virus* host preference?. Virus Genes.

[18] Valasevich N., Cieślińska M., Kolbanova E. (2015). Molecular characterization of *apple mosaic virus* isolates from apple and rose. Eur. J. Plant Pathol..

[19] Ulubaş Serçe Ç., Ertunç F., Oeztuerk A. (2009). Identification and genomic variability of prune dwarf virus variants infecting stone fruit trees in turkey. J. Phytopathol..

[20] Vašková D., Petrzik K., Špak J. (2000). Molecular variability of the capsid protein of the prune dwarf virus. Eur. J. Plant Pathol..

[21] Predajňa L., Sihelská N., Benediková D., Šoltys K., Candresse T., Glasa M. (2017). Molecular characterization of prune dwarf virus cherry isolates from slovakia shows their substantial variability and reveals recombination events in pdv RNA3. Eur. J. Plant Pathol..

[22] Constable F.E., Joyce P.A., Rodoni B.C. (2007). A survey of key australian pome fruit growing districts for exotic and endemic pathogens. Australas. Plant Pathol..

[23] Stubs L., Smith P. (1971). The association of prunus ringspot, prune dwarf, and dark green sunken mottle viruses in the rosetting and decline disease of peach. Crop Pasture Sci..

[24] Greber R., Teakle D., Mink G. (1992). Thrips-facilitated transmission of prune dwarf and *prunus necrotic ringspot viruses* from cherry pollen to cucumber. Plant Dis..

[25] Parakh D., Shamloul A., Hadidi A., Waterworth H., Scott S., Howell H., Mink G. (1994). Detection of prune dwarf *ilarvirus* from infected stone fruits using reverse transcription-polymerase chain reaction. Acta Hortic..

[26] Petrzik K., Svoboda P. (1997). Screening of *apple mosaic virus* in hop cultivars in the czech republic by reverse transcription-polymerase chain reaction. Acta Virol..

[27] Kinoti W.M., Constable F.E., Nancarrow N., Plummer K.M., Rodoni B. (2017). Analysis of intra-host genetic diversity of *prunus necrotic ringspot virus* (PNRSV) using amplicon next generation sequencing. PLoS ONE.

[28] Edgar R.C. (2004). Muscle: Multiple sequence alignment with high accuracy and high throughput. Nucleic Acids Res..

[29] Martin M. (2011). Cutadapt removes adapter sequences from high-throughput sequencing reads. EMBnet J..

[30] Stamatakis A. (2014). Raxml version 8: A tool for phylogenetic analysis and post-analysis of large phylogenies. Bioinformatics.

[31] Andrew R. Figtree. http://tree.bio.ed.ac.uk/software/.

[32] Muhire B.M., Varsani A., Martin D.P. (2014). Sdt: A virus classification tool based on pairwise sequence alignment and identity calculation. PLoS ONE.

[33] Kinoti W.M., Constable F.E., Nancarrow N., Plummer K.M., Rodoni B. (2017). Generic amplicon deep sequencing to determine *ilarvirus* species diversity in australian *prunus*. Front. Microbiol..

[34] Tamura K., Stecher G., Peterson D., Filipski A., Kumar S. (2013). Mega6: Molecular evolutionary genetics analysis version 6.0. Mol. Biol. Evol..

[35] Gümüs M., Paylan I., Matic S., Myrta A., Sipahioglu H., Erkan S. (2007). Occurrence and distribution of stone fruit viruses and viroids in commercial plantings of prunus species in western anatolia, turkey. J. Plant Pathol..

[36] Myrta A., Di Terlizzi B., Savino V., Martelli G. (2003). Virus diseases affecting the mediterranean stone fruit industry: A decade of surveys. Virus and Virus-Like Diseases of Stone Fruits, with Particular Reference to the Mediterranean Region.

[37] Rouag N., Guechi A., Matic S., Myrta A. (2008). Viruses and viroids of stone fruits in algeria. J. Plant Pathol..

[38] Petrzik K. (2005). Capsid protein sequence gene analysis of *apple mosaic virus* infecting pears. Eur. J. Plant Pathol..

[39] Kalinowska E., Mroczkowska K., Paduch-Cichal E., Chodorska M. (2014). Genetic variability among coat protein of prune dwarf virus variants from different countries and different *prunus* species. Eur. J. Plant Pathol..

[40] Cieślińska M., Valasevich N. (2016). Characterization of apple mosaic virus isolates detected in hazelnut in poland. J. Plant Dis. Prot..

[41] Boulila M. (2010). Molecular characterization of an almond isolate of prune dwarf virus in tunisia: Putative recombination breakpoints in the partial sequences of the coat protein-encoding gene in isolates from different geographic origin. Phytopathol. Mediterr..

[42] Öztürk Y., Çevik B. (2015). Genetic diversity in the coat protein genes of prune dwarf virus isolates from sweet cherry growing in turkey. Plant Pathol. J..

